# Sense and readability: participant information sheets for research studies

**DOI:** 10.1192/bjp.bp.114.156687

**Published:** 2016-02

**Authors:** Liam Ennis, Til Wykes

**Affiliations:** **Liam Ennis**, BSc, MSc, Health Services and Population Research Department, Institute of Psychiatry, Psychology and Neuroscience, King's College London; **Til Wykes**, DPhil, Psychology Department, Institute of Psychiatry, Psychology and Neuroscience, King's College London, UK

## Abstract

**Background**

Informed consent in research is partly achieved through the use of information sheets. There is a perception however that these information sheets are long and complex. The recommended reading level for patient information is grade 6, or 11–12 years old.

**Aims**

To investigate whether the readability of participant information sheets has changed over time, whether particular study characteristics are related to poorer readability and whether readability and other study characteristics are related to successful study recruitment.

**Method**

We obtained 522 information sheets from the UK National Institute for Health Research Clinical Research Network: Mental Health portfolio database and study principal investigators. Readability was assessed with the Flesch reading index and the Grade level test.

**Results**

Information sheets increased in length over the study period. The mean grade level across all information sheets was 9.8, or 15–16 years old. A high level of patient involvement was associated with more recruitment success and studies involving pharmaceutical or device interventions were the least successful. The complexity of information sheets had little bearing on successful recruitment.

**Conclusions**

Information sheets are far more complex than the recommended reading level of grade 6 for patient information. The disparity may be exacerbated by an increasing focus on legal content. Researchers would benefit from clear guidance from ethics committees on writing succinctly and accessibly and how to balance the competing legal issues with the ability of participants to understand what a study entails.

Study information sheets must contain sufficient detail for potential participants to make an informed decision about taking part. However, detail must be balanced with the competing demand of comprehension. Several studies have shown that longer information sheets can result in poorer retention and comprehension of important information than briefer versions.^1–3^ This might be because longer information sheets are less likely to be read.^4^

Previous research also indicates that information sheets are complex.^5–7^ Recent studies report the average reading grade of information sheets at around US grade 12, or age 17–18 years.^4,8–10^ This is far above the US National Institutes of Health recommended reading level for health information of grade 6, or age 11–12 years.^11^ Excessive complexity is likely to intimidate potential participants and impair comprehension. In turn, this could hamper participant recruitment.^12,13^

In a previous study, we used the National Institute for Health Research Clinical Research Network (NIHR CRN): Mental Health portfolio database to demonstrate an association between the quality of patient involvement in a study and successful recruitment.^14^ We use the same technique here to investigate whether participant information sheets have become more complex over time, and whether information sheet complexity is a result of particular study features. Finally, we investigate whether readability of information sheets is related to recruitment success.

## Method

### Sample of studies

We obtained 522 unique information sheets for non-commercially sponsored studies registered on the NIHR CRN: Mental Health portfolio database over 10 years (June 2003–October 2013). Our sample represents over 52% of all studies listed on the database at the time of writing.

We obtained the same proportion of information sheets from each area of clinical study. However, our sample was more likely to contain information sheets for intervention studies than observational studies, and this relationship was not explained by the increasing number of interventional studies over time.

The portfolio database contains information on study design, recruitment target completion and patient involvement for each study. Information sheets were obtained from principal investigators and from NIHR CRN systems.

### Measures

#### Readability scores

For each information sheet, we recorded:

- number of words
- Flesch index^15^ and reading grade^15^ for the whole sheet, with headings and subheadings removed
- Flesch index and reading grade for the most complex section.

The Flesch index and reading grade level are two widely used measures of a documents' readability. The formulas are based on sentence length, number of words and number of syllables. Higher Flesch index scores indicate greater readability, whereas higher grade level scores indicate poorer readability.

A ‘section’ was defined as any piece of text immediately following a heading consisting of at least 100 words, truncated at the first full stop after 100 words. If sections were shorter than 100 words, the next subheading was removed and counting continued into the next section. Sections excluded lists or contact details.

#### Study characteristics

We also recorded:

- Clinical study group (CSG): the clinical area to which a study belonged. These are based on the strategic analysis of UK mental health research funding categories.^16^
- Level of patient involvement in the study: based on categories reported in Ennis & Wykes.^14^ These were consultation only, researcher initiated collaboration and user controlled/user initiated collaboration.
- Study complexity: on a 1–17 scale, with higher scores indicating greater complexity, calculated on factors such as number and frequency of follow-ups, number of study sites and involvement of patients who lack capacity.
- Intervention type: whether the study included an intervention, and if so, what that was.
- Recompense value: how much payment was received for participation.
- Whether a study had or was on target to recruit to time and target. This was a binary variable, with >90% indicating successful recruitment as this usually enables a valid test of the study hypothesis.

### Statistical analysis

To assess whether information sheets had changed over 10 years, we calculated Spearman's Rho between time and the readability measures.

To identify differences in readability measures between CSGs, we used one-way analysis of variance (ANOVA). The Games–Howell procedure was used to test differences in means in the presence of heteroscedasticity.

ANOVA was also used to compare readability measures between studies with different interventions and to investigate whether it was affected by different levels of patient involvement. The Games–Howell procedure was used for *post hoc* comparisons where necessary. Mean Flesch index scores for the most complex section were compared using Hochberg's GT2.

Binary logistic regression identified associations between various predictors and successful recruitment. All putative study characteristic predictors were entered simultaneously (those identified by Ennis & Wykes,^14^ participant payment and type of intervention), along with the Flesch index score for the whole information sheet. Other readability measures were highly correlated so we chose the Flesch index because it has greater precision than the reading grade score.

#### Sample size

We used G*Power 3.1.9 for sample size calculations.

- Time×readability correlations: with a sample size of 472, we had 90% power to detect a correlation of 0.1, one-sided (α = 0.05).
- Readability differences between CSGs: with a sample size of 37 per group, we had 90% power to detect an effect size of 0.3, two-sided (α = 0.05).
- Readability differences between intervention types: with a sample size of 20 per group, we had 90% power to detect an effect size of 0.4, two-sided (α = 0.05).
- Readability differences between levels of patient involvement: with a sample size of 120 per group, we had 90% power to detect an effect size of 0.2, two-sided (α = 0.05).
- Associations with successful recruitment: with a sample size of 313, we had 90% power to detect an odds ratio of 1.5, two-sided (α = 0.05).

## Results

A total of 278 of 522 (53%) studies were observational. A total of 254 studies (48.7%) offered some recompense for participation. Studies that involved psychotic disorders or addictions were more likely to provide payment than studies in other CSGs (χ^2^(4) = 47.60, *P*<0.001) (Table 1).

**Table 1:** Study characteristics

Intervention type,^a^ *n* (%)	
Psychological	157 (30)
Pharmacological/device	41 (8)
Service	26 (5)
Other	20 (4)
Observational	278 (53)

Study complexity,^b^ mean (s.d.)	9.59 (4)

Clinical study group,^c^ *n* (%)	
Psychotic disorders	130 (25)
Mood and personality disorders	179 (34)
Services research	80 (15)
Dementias and intellectual disability	76 (15)
Addictions	37 (7)

Patient involvement,^d^ *n* (%)	
Consultation	200 (38)
Researcher initiated collaboration	199 (38)
Jointly/patient initiated collaboration/patient control study	120 (23)

Recompense value,^c^ £	
Median	0.00
Median for those providing compensation	30.00
Range	5–300

Readability statistics,^a^ mean (s.d.)	
Number of words	1527 (821)
Flesch index: whole sheet	58.79 (9)
Grade level: whole sheet	9.84 (2)
Flesch index: most complex section	41.50 (13)
Grade level: most complex section	13.33 (3)

a. *n* = 522.

b. *n* = 520.

c. *n* = 502.

d. *n* = 519.

The mean number of words in an information sheet was 1527, but length varied widely (range 161–5407). The mean Flesch index score was 59 (range 29.2–92.0) for whole information sheets, grade level 10 (range 3–14) or 15–16 years old. For the most complex section, the mean Flesch index score was 42 (range 1.8–84.2), grade level 13 (range 5–27) or 18–19 years old.

### Have information sheets become more complex over time?

Information sheets significantly increased in length over 10 years (ρ = 0.18, *P*<0.001). The increase is from an average of 1333 words in 2003 to 1714 words in 2013. This relationship was not explained by an increasing number of interventional studies over time. Despite increases in length, there was no change over time in Flesch reading index or grade level.

### Do study characteristics affect readability?

Information sheets from different CSGs varied significantly in length as measured by number of words (*F*_(4, 497)_ = 9.80, *P*<0.001), the Flesch index for the most complex section (*F*_(4, 497)_ = 5.42, *P*<0.001) and the whole information sheet (*F*_(4, 497)_ = 8.10, *P*<0.001). The mean scores for these measures as a function of CSG are presented in Table 2.

**Table 2:** Mean readability measures for different clinical study groups (CSG), intervention types and levels of patient involvement^a^

		Flesch reading index		Reading grade level	
	Words, *n*	Whole sheet	Most complex section	Whole sheet	Most complex section
CSG					
Dementia and intellectual disability	1297 (1155–1440)	63.59 (60.69–66.50)	47.67 (43.75–51.60)	8.76 (8.19–9.34)	11.77 (10.96–12.59)
Mood and personality disorders	1649 (1521–1777)	58.24 (57.01–59.47)	39.43 (37.40–41.46)	10.03 (9.78–10.28)	13.75 (13.26–14.23)
Services research	1176 (1079–1273)	56.65 (55.09–58.21)	41.78 (39.38–44.17)	10.26 (9.97–10.56)	13.34 (12.83–13.85)
Psychotic disorders	1590 (1456–1724)	58.24 (57.17–59.31)	40.61 (38.63–42.59)	9.93 (9.72–10.14)	13.59 (13.08–14.10)
Addictions	2002 (1547–2457)	57.65 (55.57–59.73)	40.80 (36.81–44.79)	9.99 (9.56–10.42)	13.32 (12.35–14.29)

Intervention type					
Observational	1267 (1200–1333)	58.48 (57.49–59.47)	41.86 (40.37–43.40)	9.84 (9.64–10.04)	13.25 (12.90–13.61)
Psychological	1547 (1450–1645)	59.75 (58.31–61.19)	42.88 (40.58–45.18)	9.74 (9.45–10.03)	13.16 (12.64–13.69)
Pharmacological/device	2898 (2514–3282)	56.20 (53.84–58.56)	35.36 (31.58–39.14)	10.41 (9.98–10.84)	14.04 (13.25–14.83)
Service	1410 (1176–1645)	59.80 (56.89–62.70)	41.94 (37.29–46.59)	9.82 (9.38–10.25)	14.02 (12.39–15.66)
Other	2318 (1745–2892)	59.54 (55.02–64.05)	37.52 (30.20–44.84)	9.58 (8.69–10.46)	13.34 (13.06–13.60)

Patient involvement					
Consultation	1643 (1510–1775)	59.05 (57.79–60.30)	41.62 (39.77–43.48)	9.73 (9.49–9.97)	13.08 (12.66–13.50)
Researcher initiated collaboration	1509 (1400–1617)	58.73 (57.46–59.99)	41.67 (39.76–43.57)	9.87 (9.61–10.12)	13.32 (12.88–13.77)
Jointly/patient initiated collaboration/ patient control study	1355 (1243–1467)	58.33 (57.04–59.63)	41.09 (38.70–43.49)	10.00 (9.74–10.26)	13.67 (13.12–14.22)

a. 95% confidence intervals are shown in parentheses.

Information sheets for studies involving dementias and intellectual disabilities were the shortest and easiest to read of any CSG.

Information sheets for studies including different interventions varied in length (*F*_(4, 517)_ = 58.00, *P*<0.001) and in Flesch index scores for the most complex section (*F*_(4, 517)_ = 3.12, *P* = 0.015). Studies including a pharmacological or device intervention were the longest and most complex. Unsurprisingly, information sheets for studies containing no intervention (i.e. observational studies) were the shortest. There was no significant difference on the Flesch index scores for the whole information sheet. The mean scores for all of these measures are presented in Table 2.

The length of information sheets was significantly different between studies which included different levels of patient involvement (*F*_(2, 516)_ = 4.71, *P* = 0.009). This was an inverse linear relationship; as patient involvement increased, length decreased by about 150 words. There was no significant difference in the other readability measures. Means are given in Table 2.

### Does readability predict recruitment success?

The equation used to identify associations with successful recruitment was very close to statistical significance (deviance χ^2^ (14) = 23.19, *P* = 0.057). The model (Table 3) produced a Nagelkerke pseudo-*R*^2^ of 0.064.

**Table 3:** Logistic regression showing associations with successful recruitment

	Beta	Odds ratio	95% CI
Clinical study group			
Dementia and intellectual disability		Reference	
Mood and personality disorders	−0.04	0.96	0.52–1.76
Services research	−0.37	0.69	0.34–1.42
Psychotic disorders	−0.10	0.90	0.48–1.70
Addictions	0.44	1.56	0.63–3.83

Complexity	−0.02	0.99	0.94–1.04

Patient involvement			
Consultation only		Reference	
Researcher initiated collaboration	0.24	1.28	0.83–1.97
Patient initiated or patient controlled	−**0.51**	**1.66**	1.00–2.77*

Intervention type			
Observational		Reference	
Psychological	−0.19	0.83	0.51–1.34
Pharmacological/device	−**1.44**	**0.24**	**0.10**–0.54**
Service	0.12	1.13	0.45–2.84
Other	−0.50	0.61	0.22–1.70

Flesch index (whole sheet)	0.00	1.00	0.98–1.02

Recompense value	0.05	1.01	1.00–1.01

Opening date	0.00	1.00	1.00–1.00

Significant associations are in bold.

* *P*<0.05.

** *P*<0.001.

The model shows that studies which involved a pharmacological or device intervention were less likely to recruit to time and target than other types of study. Studies that involved patients at the highest level were more likely to achieve successful recruitment, but readability did not contribute to recruitment success.

## Discussion

We have analysed a study sample nearly double the size of the next largest study (284 studies).^17,18^ We also covered the longest period, 10 years. This has allowed us to reveal some important information on the state of participant information sheets for today's research studies.

### Have information sheets changed over time?

Our data demonstrate that information sheets have grown longer over time. This may be a result of the increasing focus on patient safety – what some might term risk aversion – over the period of study.^19,20^ This is noteworthy because previous research found that more detailed information sheets are less well understood than briefer versions.^1–3^ Longer information sheets are also less likely to be read.^2,4^ Taken with these findings, our results imply that participants' understanding may have actually decreased over time. This is a hypothesis that needs testing, as it has implications for ethics committee advice. On the positive side, information sheets do not appear to have become more complex over time.

### Is readability affected by study characteristics?

Information sheets for studies investigating dementias and intellectual disabilities were easier to read than studies in some other areas. This is unsurprising, since many studies belonging to this CSG used simplified language supported with lots of pictures. However, even with this adjustment, information sheets in this CSG only crept into the ‘standard’ range of reading difficulty (Flesch index score 61–70),^15^ requiring an estimated reading grade of 9 or 13–14 years old. Information sheets for all other CSGs were firmly in the ‘fairly difficult’ category (Flesch index score 51–60). In addition, the mean score for the most complex section in every CSG fell into the ‘difficult’ category (Flesch index score 31–50).

Our data also show that the information sheets for observational studies were significantly shorter than some other types. This is unsurprising since observational studies need not describe interventions, the process of group allocation or masking procedures. Perhaps more interesting is the average length of information sheets for studies including a pharmacological or device intervention – a staggering 3000 words. Pharmacological and device intervention studies also performed poorly with regard to the most complex section, with the average score falling into the ‘very difficult’ category (0–30). Texts scoring in this range are comparable to scientific writings.^15^ This is of particular importance since more complex information sheets tend to accompany studies which carry the most risk.^21^

Higher levels of patient involvement seem to facilitate briefer information sheets. This could be a product of patients reviewing information sheets and commenting on sections which could be shortened. It is interesting to note, however, that patient involvement did not seem to mitigate against complex information sheets overall nor against very dense sections of text.

Collapsing the groups used in this study, the average whole-sheet Flesch index score was 59, corresponding to US grade 10, or a reading age of 15–16 years old. This is higher than the UK national reading age of US grade 8, or 13–14 years old.^22^ It is also markedly higher than the recommended reading age for patient information texts (US grade 6, or 10–11 years). About 89% of the information sheets in our sample were at or above the national reading age, and 96% were at or above the recommended age for patient information texts.

The most complex sections of information sheets were very demanding. On average they scored 42 on the Flesch index, which corresponds to a reading age of 18–19. One information sheet we analysed scored 73 (fairly easy) for the whole sheet but a dismal 12 (very difficult) for the most complex section, showing how important it is to consider the complex section in any analysis.

Despite the poor performance of the information sheets we analysed, the results were better than those reported by others. A study of mental health research recorded a mean Flesch index score of 48, or grade 12.^8^ Similarly, oncology studies scored 45,^4^ anaesthesia research a reading grade 12,^10^ and one cross-discipline French study actually reported a median Flesch index score of 24 (very difficult).^4^ We have examined the methods of these studies and they are comparable to our own. The different results might therefore be attributed to variation in the composition of study samples. It seems likely, for example, that a sample of anaesthesia studies would contain more clinical trials than our own sample.

There are a number of techniques to reduce the reading age of information sheets which are obvious – using shorter words, sentences and paragraphs and replacing complex medical and research terms with simple words.^23^ Unfortunately, the UK Health Research Authority (HRA) in the UK issue only vague guidance on the drafting of information sheets.^24^ For example, they unhelpfully suggest that ‘A participant information sheet should be as long as it needs to be’. Nor do the HRA recommend a particular Flesch index range, although they do advocate use of the measure to ‘help improve readability of your information sheet’. The University of Michigan already provides a guide for simplifying medical terms.^25^ A similar glossary could be produced to include research terms such as ‘randomisation’ and ‘double-blind’. In the Appendix, we demonstrate that it is possible to dramatically improve the readability of complex passages without extending – and in some cases reducing – overall length. In one case, we were able to increase the Flesch index score from 18.9 (very difficult) to 73.6 (fairly easy).

### Does readability have implications for recruitment?

Our analysis showed that the complexity of information sheets had little bearing on successful recruitment. The odds ratio of one indicates that even very large changes in Flesch index score are unlikely to affect the chances of recruitment success. Our results therefore suggest that information sheet complexity, at least as measured by the Flesch index, remains an ethical problem rather than an ingredient for study success.

We found that studies including a pharmacological or device intervention were less likely to have reached their target than observational studies even in this non-commercial funded dataset. Overall, however, this finding will not come as a surprise to many readers: difficulty in recruiting to drug trials is an enduring issue.^26–29^ Qualitative studies have shown that some people dislike the idea of ‘being a guinea pig’,^30^ the rigidity of treatment regimes and the prospect of side-effects.^31^

Studies which included the highest level of patient involvement were more likely to have reached their recruitment target. This finding replicates our earlier work with a much larger sample,^14^ which provides further evidence of the importance of this factor in recruitment success.

### Limitations

There are two limitations to this study. The first is that the study did not investigate comprehension by real people but instead used an analogue – the Flesch index. Although the Flesch index is an accepted proxy for reading difficulty, actual comprehension in terms of a research study is impossible to capture with a formula. Information sheets are typically augmented by conversations with research or clinical staff at the point of consent. However, the information sheet is what the potential participant can take away from this encounter and if they do not understand it then there are clearly ethical implications. The second limitation concerns our findings relating to recruitment success. We have not conducted a randomised controlled trial, and therefore we cannot rule out confounding. We have tried to capture many different variables that are thought to be important in the largest observational study to date. Further information from qualitative or randomised studies would be helpful.

### Policy and ethics implications

Information sheets are approved by ethics committees, and yet they tend to consist of text written at a level far higher than can be assumed of the average reader. Information sheets are also becoming longer, and this may further impede participants' understanding.

Some have suggested that their length and complexity are increasing because ethics committees and principal investigators now emphasise the legal, rather than communicative, aspects.^4^ In our study, the most complex section was often standardised content such as insurance arrangements or confidentiality policies. Other investigators have found that attempts to simplify these template passages are rejected because of concerns of how text alterations might affect the legality of the statements.^32^ Clearly this creates an impasse whereby important legal text will never be understood. If this is the case, fear of litigation has distorted the real purpose of study information sheets, leaving review boards protected but participants uninformed.

Principal investigators and ethics committees must balance legal and ethical issues and we consider that this is now out of kilter. We therefore suggest that they critically review their standardised content. It is ethically unsound to allow such text to be included when it is clear that to understand it participants would need their own lawyers to provide an explanation.

Clearly, there is some way to go before information sheets are written to a standard which is likely to be understood by most potential participants. We did find that the information sheets we analysed were considerably easier to read than in some other recent studies, but this provides little solace as they were generally still much more complex than the recommended grade 6 level. Principal investigators and ethics committees must consider the length, complexity, and – most importantly – the purpose of information sheets if this standard is to be achieved.

## Appendix

### (a) Confidentiality

#### How will confidentiality and anonymity be ensured?

All your questionnaire responses will be totally confidential: published reports will not allow the responses of any individual worker to be identified in any way, nor will any information about individual responses be fed back to colleagues or managers in your service. The Response Confirmation sheet that you have been given with the questionnaire has a study number on it: only the local research worker knows which number belongs to which staff member, and we have used these numbers only to allow the researcher to check which staff members have returned the questionnaire and which have not.

This section has a Flesch index score of 18.9, which is in the same region as many scientific writings. It is 97 words long.

#### Will anyone know I've taken part?

We won't tell your manager or anyone else that you have taken part. We also won't include your name, or anything else which could identify you, in anything we publish.

We will keep your answers separated from your name. We replace names with numbers so that no-one will know what answers you gave.

We have rewritten this section and its Flesch index score is now 73.6 (fairly easy). It is now 53 words long.

### (b) Invitation to take part

#### Invitation to take part in our study

The test we have just carried out has suggested you are suffering from significant health anxiety, excessive concern about your health which is causing you problems. We think we now have ways of helping people with this problem and so you are being invited to take part in what is called a randomised control trial, in which you would be allocated one of two treatments, either a treatment called ‘cognitive behavioural therapy’ which will be given for between 5 and 10 sessions of just under an hour each time, or a simple explanation of what health anxiety is and how it affects people.

*This section has a Flesch index score of 28.8, which again falls into the ‘very difficult’ range of scores. It is 103 words long*.

#### Invitation to take part in our study

The test you just did makes us think that you are very worried about your health. We have a new treatment which might help people who worry about their health. The treatment is called cognitive behavioural therapy. We need to know if the treatment works and so we are doing an experiment. In the experiment, we will compare two groups of people. One group will receive cognitive behavioural therapy for 5–10 sessions. Each session will last an hour. The other group will simply receive some information about health worries. The group people are in will be decided at random. Would you like to know more?

*Our version has a Flesch index score of 75. It is 107 words long*.
